# Study on the Performance of a Novel Microbial-Assisted Chemical Viscosity Reduction Technology for Enhancing Heavy Oil Displacement Efficiency

**DOI:** 10.3390/molecules31071212

**Published:** 2026-04-07

**Authors:** Fan Zhang, Qun Zhang, Zhaohui Zhou, Yangnan Shangguan, Wenfeng Song, Yawen Zhou, Huilin Wang, Qianqian Tian, Kang Tang, Lei Liu

**Affiliations:** 1 Research Institute of Petroleum Exploration & Development, Beijing 100083, China; zhangfan902@petrochina.com.cn (F.Z.);; 2 Exploration and Development Research Institute of PetroChina Changqing Oilfield Company, Xi’an 710016, China; 3 School of Light Industry Science and Engineering, Beijing Technology and Business University, Beijing 100048, China

**Keywords:** enhanced oil recovery, chemical microbial, viscosity reduction efficiency, stripping oil film

## Abstract

High-viscosity reservoirs are widely distributed across various countries with abundant reserves. However, their high resin and asphaltene content leads to elevated oil viscosity and low recovery rates. Conventional chemical flooding techniques are unsuitable for the development of such high-viscosity oilfields. Chemical viscosity reduction technologies face challenges such as low viscosity reduction efficiency, poor economic feasibility, and unclear mechanisms. Microbial-assisted chemical viscosity reduction represents a relatively novel approach. This study systematically investigated the enhanced oil recovery performance of a microbial-assisted chemical viscosity reducer. The results demonstrated that this microbial-assisted chemical viscosity reducer achieved a viscosity reduction rate exceeding 85% for five different crude oil samples. It effectively altered the wettability of oil-wet surfaces, improved the oil film stripping rate by 50–65% compared to pure chemical flooding agents, and achieved ultra-low oil–water interfacial tension on the order of 10^−3^ mN/m with crude oil, leading to an enhanced oil recovery (EOR) enhancement of 22–26%. The underlying mechanism is that viscosity-reducing bacteria degrade asphaltene in heavy oil, thereby weakening intermolecular forces. Their metabolites enhance the emulsion stability of the chemical viscosity reduction process. Chemical viscosity reducers enhance the physiological cycle and metabolic activity of microorganisms while also emulsifying and dispersing heavy oil and improving emulsion stability. Therefore, this novel microbial-assisted chemical viscosity reduction technology offers a new and effective EOR method for high-viscosity reservoirs.

## 1. Introduction

With the continuous development of conventional oil fields, unconventional oil fields such as heavy oil reservoirs have become increasingly important oil and gas resources in the global petroleum industry [1,2]. High-viscosity oil reservoirs are rich in reserves and widely distributed in various countries, particularly in the United States, Canada, China, and Indonesia [3]. However, their high content of colloids and asphaltenes results in elevated crude oil viscosity and low recovery rates. Currently, the water flooding recovery rate of high-viscosity oil reservoirs is only approximately 15% [4]. Therefore, it is essential to develop effective exploitation techniques.

Conventional chemical flooding techniques enhance the recovery rate of low-viscosity oil reservoirs by modifying the properties of the aqueous phase, thereby expanding the swept volume and improving oil displacement efficiency. Typical chemical viscosity reduction techniques include surfactant/alkali and surfactant/polymer (SP) flooding technologies. Surfactants can reduce the interfacial tension (IFT) between oil and water and emulsify the oil into water-in-oil (O/W) emulsions, thereby decreasing oil viscosity and improving oil mobility [5,6]. Surfactants, alkalis, and their combined flooding have been successfully employed as cost-effective non-thermal recovery techniques to increase heavy oil recovery rates [7,8,9]. However, due to issues such as alkali consumption, severe scaling, and accelerated pipeline corrosion, the on-site application of alkalis has gradually been limited [10,11]. Surfactant/polymer (SP) flooding technology not only exhibit characteristics such as low IFT and strong emulsification ability for heavy oil, but also demonstrate advantages such as high injected fluid viscosity and rapid reduction of oil–water ratio, thereby simultaneously improving microscopic displacement efficiency and macroscopic swept volume [12,13]. However, commonly used polymers in the SP system, such as polyacrylamide (PAM), partially hydrolyzed polyacrylamide (HPAM), and their derivatives, form precipitates when combined with Ca^2+^ and Mg^2+^ ions under high-salinity conditions, which diminishes the effectiveness of heavy oil recovery. Therefore, chemical viscosity reduction technologies still face challenges, including a low viscosity reduction rate, poor cost-effectiveness, and unclear mechanisms, rendering them unsuitable for the development of high-viscosity oil fields.

Resin and asphaltene are components of heavy oil with high polarity and high molecular weight, and they are the main reasons for the high viscosity and poor degradability of heavy oil [14]. Currently, there are few studies on specific microbial strains capable of degrading resins and asphaltenes, and in particular, the degradation efficiency of such strains toward these components remains extremely low [15]. For instance, Lavania et al. [16] employed Garciaella petrolearia (TERIG02) to degrade asphaltenes, reducing the average molecular weight of the crude oil and thereby lowering its viscosity. Shahebrahimi et al. [17] isolated indigenous bacteria from crude oil samples and used them to biodegrade asphaltenes, achieving a maximum asphaltene degradation rate of 41.95%, with a significant reduction in the contents of carbon, hydrogen, and nitrogen. Gao et al. [18] utilized Pseudomonas aeruginosa to degrade refractory asphaltenes in crude oil, converting heavy components into light fractions. Pourfakhraei et al. [19] treated heavy oil with *Daedaleopsis* sp. and found that a decrease in the content of heavy components (C24+) and an increase in light components (C24−). Microbial viscosity reduction technology offers advantages such as low cost, absence of pollution, and ease of fluid handling. The technology is well-suited for heavy oil with high water content and a low recovery rate, significantly improving the recovery rate [20,21,22,23]. However, microbial viscosity reduction technology has some limitations, including long cycles, low efficiency, and difficulties in microbial cultivation and screening [24]. Additionally, it has been found that heavy oil is typically under conditions of high salinity, high temperature, and high metal ion content, which are unfavorable for the survival of microorganisms [25,26,27]. Therefore, microbial viscosity reduction technology is still in the exploratory stage.

Microbial-assisted chemical viscosity reduction is an emerging technology that remains largely unexplored in the literature [28]. This paper carried out a detailed investigation of the performance of a microbial-assisted chemical viscosity reducer, including the interaction between viscosity-reducing bacteria and chemical agents, viscosity reduction efficiency, wettability alteration capability, interfacial properties, oil displacement efficiency, and simulated analysis of the viscosity reduction mechanism. The research provides new insights into effective EOR methods for high-viscosity reservoirs.

## 2. Results and Discussion

### 2.1. Interaction Between Microbial Bacteria and Chemical Agents

**Figure 1:**
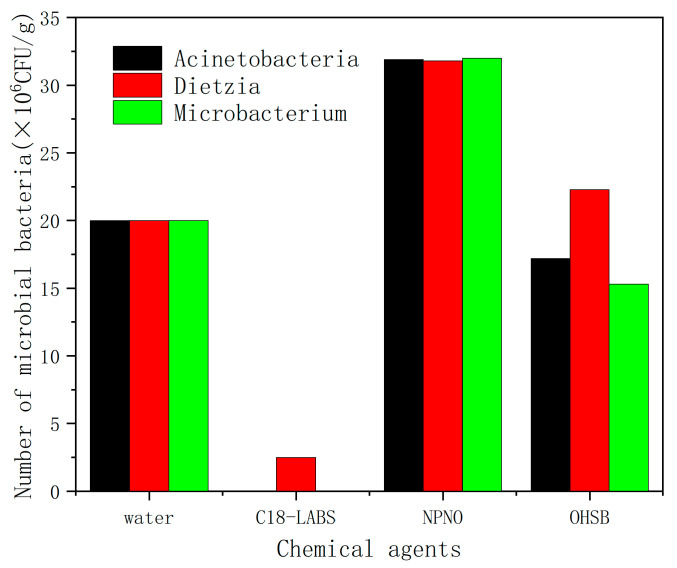
The compatibility experiment results of viscosity-reducing bacteria and chemical agents.

The interactions between three chemical viscosity reducers and three viscosity-reducing bacteria were studied using the plate colony counting method. The compatibility test results are shown in Figure 1. The results indicated that C_18_ linear alkylbenzene sulphonate (C18-LABS) significantly reduced the bacterial counts of all three viscosity-reducing bacteria to very low levels. For example, the bacterial count in the Dietzia system had a maximum of only 2.5 × 10^6^ CFU/g, and the bacterial count in the Acinetobacteria system was reduced to nearly zero. Therefore, C18-LABS significantly inhibited the activity of all three viscosity-reducing bacteria. The reason may be that C18-LABS disrupts the cell membrane structure of viscosity-reducing bacteria, leading to leakage of intracellular contents, thereby inhibiting or even killing the cells. Alternatively, the surfactant may alter the osmotic pressure and ionic strength of the solution beyond the tolerance range of the bacteria, inhibiting their growth. N-Polyoxyethylated N-octadecylamine (NPNO) significantly increased the bacterial counts in all three viscosity-reducing bacterial systems, reaching approximately 31 × 10^6^ CFU/g. NPNO appeared to be utilized as a carbon source by the bacteria and promoted their growth. Thus, the three viscosity-reducing bacteria showed significant degradation capability to NPNO. As can be seen in Figure 1, the bacterial counts in oleic acid amidopropyl hydroxysulfonyl betaine (OHSB) in the three viscosity-reducing bacteria systems were not significantly different from that in the pure water system with viscosity-reducing bacteria, and the bacterial count changed little. Therefore, OHSB exhibited good compatibility with three viscosity-reducing bacteria. Based on these results, OHSB was selected for subsequent studies.

### 2.2. Viscosity Reduction Results of Viscosity-Reducing Bacteria

The initial viscosity of the crude oil used in the experiment was 64.4 mPa·s, and the concentration of OHSB was 0.30 wt%. The viscosity reduction performance of three kinds of microbial bacteria was studied and the viscosity reduction results are shown in Table 1. It can be seen that Microbacterium showed the lowest viscosity after viscosity reduction and the highest reduction rate of 85.2%. Therefore, Microbacterium was selected for subsequent experiments.

### 2.3. Viscosity Reduction Capability for Different Oils

A microbial-assisted chemical viscosity reducer system composed of 0.30 wt% OHSB and Microbacterium was tested for its viscosity reduction capability on five different oil samples at their respective reservoir temperatures.

The experimental results are listed in Table 2. It can be seen that the viscosity reduction efficiency of the viscosity reducer was more than 85%. Thus, the microbial-assisted chemical viscosity reducer system had good viscosity reduction capability for different oils.

### 2.4. Wettability Alteration Capacity for Different Oils

The wetting alteration capacity of the microbial-assisted chemical viscosity reducer system composed of 0.30 wt% OHSB and Microbacterium was tested on five different crude oils at their respective reservoir temperatures.

It can be seen in Table 3 that the oil film stripping time of the chemical surfactant system for the five crude oil samples was between 23 and 125 s, while the oil film stripping time of the microbial-assisted chemical viscosity reducer system for the five crude oil samples was between 9 and 56 s. Compared with the chemical surfactant system, the reduction rate in oil film stripping time in the microbial-assisted chemical viscosity reducer system was 52–63%. Therefore, the microbial-assisted chemical viscosity reducer system had a stronger ability to alter wettability, which can quickly peel off oil films and facilitate faster oil recovery rates.

### 2.5. Interfacial Tension (IFT) Analysis

The IFT between oil and water under the microbial-assisted chemical viscosity reducer system composed of 0.30 wt% OHSB and Microbacterium were investigated. The IFT results are shown in Table 4. The IFT values using the chemical surfactant system were in the range of 2.3 × 10^−2^ to 4.1 × 10^−2^ mN/m, while the IFT of those using the chemical and microbial composite system were in the range of 1.9 × 10^−3^ to 3.6 × 10^−3^ mN/m. The microbial-assisted chemical viscosity reducer system decreased IFT by one order of magnitude. Therefore, the microbial-assisted chemical viscosity reducer had better interfacial capability.

### 2.6. Simulation Calculation of Enhanced Oil Recovery and Oil Displacement Efficiency

**Figure 2:**
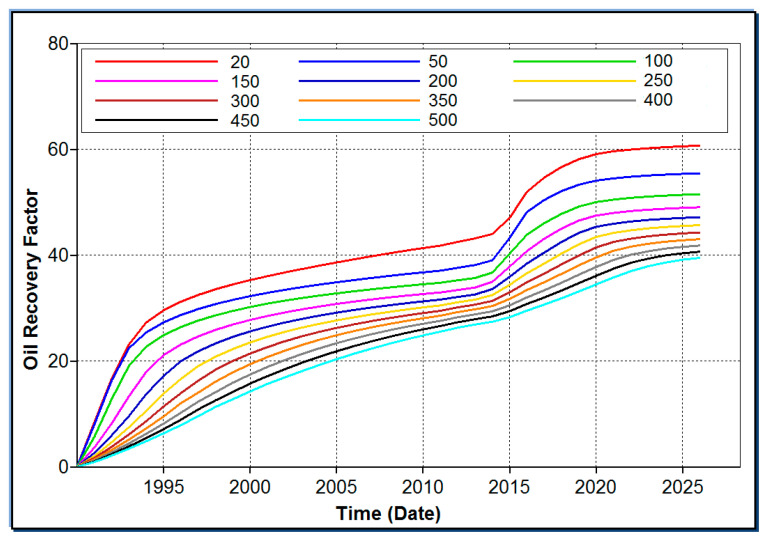
EOR results of different oil viscosity systems.

A significant reduction in crude oil viscosity can substantially improve the flowability of the oil phase, and is one of the most effective measures to optimize the flowability ratio of the displacing phase to the displaced phase. The results of CMG numerical simulation calculation are shown in Figure 2. It can be seen that when the viscosity of crude oil was in the range of 20 to 500 mPa·s, viscosity reduction flooding increased the recovery rate by about 20% on the basis of water flooding.

Core flooding experiments were conducted to evaluate the potential for improving oil recovery by reducing viscosity in different systems. The initial viscosity of crude oil was 64.4 mPa·s. The concentration of OHSB and Microbacterium were both 0.30 wt%. The experimental results are listed in Table 5. The recovery of the chemical system by viscosity reduction flooding was 15.31% (Original Oil in Place, (OOIP)) after water flooding. The recovery of the microbial system was 11.89% (OOIP) after water flooding. The oil displacement proved that the microbial-assisted chemical viscosity reducer system increased oil recovery from 22.67% to 25.31% after water flooding. Therefore, the microbial-assisted chemical viscosity reducer system had better oil displacement performance, and increased oil recovery by 7.36–10% or 10.78–13.42%, respectively, compared to single chemical agents or viscosity-reducing microbial bacteria. Therefore, the microbial-assisted chemical viscosity reducer system had higher oil displacement efficiency.

### 2.7. Analysis of Viscosity Reduction Mechanism

#### 2.7.1. Key Material Basis Affecting Viscosity in Heavy Oil

**Figure 3:**
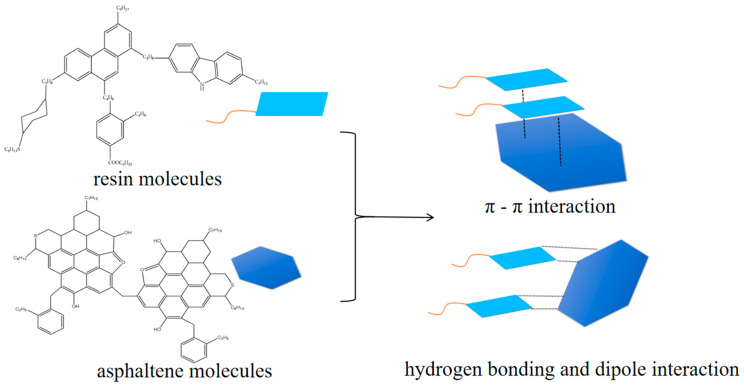
Typical structure and interaction diagram of resin and asphaltene molecules.

In heavy oil, resin and asphaltene have complex molecular structures, as shown in Figure 3. From Figure 3, it can be seen that due to the complex molecular structures of resin and asphaltene, which contain many heteroatoms and have a condensed ring structure, there are π–π interactions between aromatic rings and hydrogen bonding and dipole interactions between substituents, which result in high viscosity of heavy oil and are key factors in the formation of high-viscosity crude oil [14].

#### 2.7.2. Structure and Energy of Interaction Between OHSB with Resin and Asphaltene Dimers

**Figure 4:**
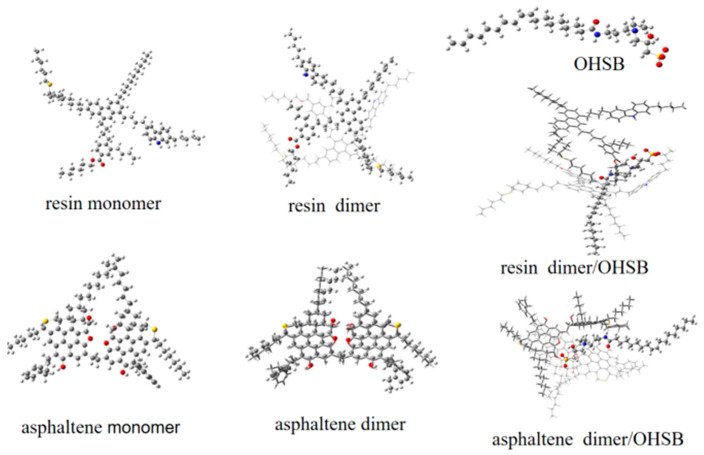
Structural diagram of the interaction between OHSB and resin/asphaltene.

In this section, the structure and energy of interaction of OHSB with resin and asphaltene molecules were analyzed using molecular simulation. The stable structures of resin and asphaltene monomers and dimers, OHSB, and the OHSB–resin/asphaltene complexes, calculated using Gaussian 09, are shown in Figure 4.

Table 6 presents the calculated energy results for resin monomer, resin dimer, asphaltene monomer, and asphaltene dimer. The results showed that the binding energy for the resin dimer was 4.99 kcal/mol, meaning the energy of the system decreased by 4.99 kcal/mol after dimerization. The energy of the asphaltene system decreased by 15.60 kcal/mol after dimerization, and was 10.61 kcal/mol lower than that of the resin dimer. This is attributed to stronger π–π and hydrogen bonding interactions. Therefore, OHSB molecules were expected to primarily interact with these dimer structures. OHSB interacted with resin and asphaltene dimers via an intercalation mechanism, as shown in Figure 4. The data in Table 4 indicate that the binding energies for OHSB intercalating into resin and asphaltene dimers were 15.88 kcal/mol and 33.02 kcal/mol, respectively. The decrease in binding energy between OHSB and the asphaltene dimer was greater than that between OHSB and the resin dimer. Therefore, OHSB primarily associated with the asphaltene dimer.

#### 2.7.3. Synergistic Viscosity Reduction Mechanism of Microbacterium and OHSB

**Figure 5:**
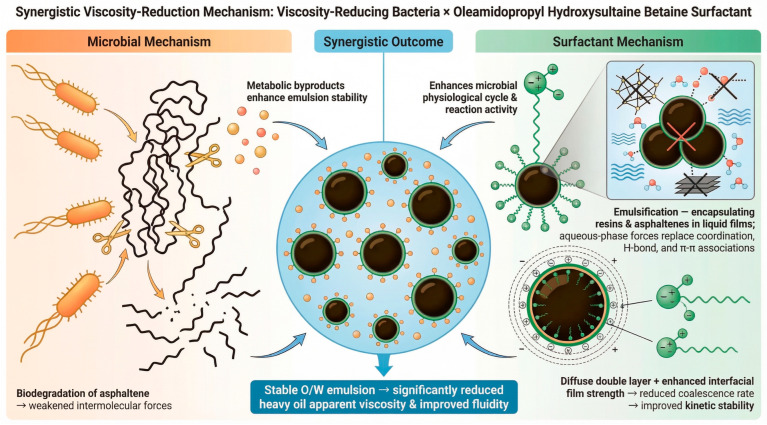
Schematic diagram of the synergistic viscosity reduction mechanism of Microbacterium and OHSB.

The viscosity reduction effect of Microbacterium manifests in two main aspects. Firstly, it can degrade asphaltene in heavy oil, and weaken inter-molecular forces [14,17,18,19,29]; secondly, its metabolic products enhance the emulsifying stability of OHSB. The viscosity reduction mechanism of OHSB operates in three ways. Firstly, it enhances the physiological cycle and metabolic activity of the microorganisms. Secondly, as a surfactant, it disperses and emulsifies the heavy oil for viscosity reduction, inserting itself into resin and asphaltene dimers, particularly asphaltene dimers. Then, it encapsulates high-viscosity resins and asphaltenes within a liquid film, and strong hydrophobic and intermolecular interactions replace the coordination, hydrogen bonding, and π–π associations among the heavy components of the crude oil. Thirdly, it reduces the coalescence rate of emulsion droplets by forming a diffuse electric double layer and enhancing the interfacial film strength through intermolecular forces, thus improving the kinetic stability of the emulsion and forming relatively stable oil-in-water emulsions that reduce the mobility of high-viscosity crude oil [30,31]. Therefore, the synergistic viscosity reduction mechanism of Microbacterium and the amphoteric surfactant OHSB is illustrated in Figure 5. It is precisely the organic synergy of these actions that endows the combination of Microbacterium and OHSB with excellent viscosity reduction performance.

## 3. Materials and Methods

### 3.1. Reagents

Five crude oil samples from different oilfields were selected. Their viscosities, origins, and reservoir temperatures are shown in Table 7. Five brine samples from the respective oilfields were used. All brines were filtered through a 0.2 μm filter before they were used for tests. The chemical surfactants were C_18_ linear alkylbenzene sulphonate (C18-LABS), N-Polyoxyethylated-N-octadecylamine (NPNO) and oleic acid amidopropyl hydroxysulfonyl betaine (OHSB), which were all purified to chemical purity before use. Microbial bacteria were Acinetobacteria, Dietzia, and Microbacterium. They were extracted from different oil fields and cultured. Both surfactants and microbial bacterium are prepared by the State Key Laboratory for Enhanced Oil Recovery of China National Petroleum Corporation (Beijing, China).

### 3.2. Experimental Method and Instruments

(1) Microbial Cultivation

Total microbial community DNA was extracted from oil reservoir water samples using the Invitrogen DNA Extraction Kit, and three indigenous strains were isolated. Corresponding clonal colonies were inoculated for separate cultivation. A standard three-stage purification process was adopted: ① Solid-state colony streaking culture on PC agar medium (composition: 0.5% peptone, 0.25% yeast extract, 0.1% glucose, 1.5% agar) was performed under static conditions at 30 °C for 24–48 h. ② A single colony was picked and inoculated into 60 mL of Nutrient Broth (composition: 1% peptone, 0.5% sodium chloride, 1% beef extract) and cultured in a shaker at 30 °C and 150 rpm for 16–18 h. ③ An aliquot of 500 μL of the bacterial culture was transferred to 500 mL of LB liquid medium and cultured in a shaker at 50 °C and 150 rpm for 36 h.

(2) Compatibility Test

The plate colony count method was employed to investigate the mutual influence between chemical viscosity reducers and viscosity-reducing bacteria by assessing the lethality rate of chemical agents on biological cells. In brief, the operational steps were as follows: The viscosity-reducing bacteria were mixed with the chemical agent in equal proportions and incubated for 48 h. Subsequently, serial dilutions were performed. One milliliter of the diluted solution was pipetted onto a pre-prepared solid plate and evenly spread using a sterile spreading rod. After the agar solidified, the plates were inverted and incubated in an incubator at 36 ± 1 °C for 48 h. Plates with colony counts ranging from 30 to 300 were selected for enumeration. Finally, the bacterial count in the original sample was calculated based on the dilution factor and the inoculation volume.

(3) Viscosity reduction rate

Viscosity reduction rate is the difference between the viscosity of the viscosity reducer emulsion and crude oil viscosity, and then the percentage of the viscosity of crude oil. The emulsion viscosity was measured with a Brookfield LVDV-IIIU viscosimeter (LVDV-IIIU, Brookfield, Middleboro, MA, USA). The experimental operation process was as follows: the mixture systems of crude oil and viscosity reducer were added to 25 mL bottles according to the different oil–water ratios. After emulsifying with homogenizer at the speed of 10,000 r/min for 1 min, 15 mL of emulsion was added to the viscosimeter [32]. The viscosity was measured at the speed of 6 rpm and the measurement results were recorded to calculate the viscosity reduction rate. The formula of the viscosity reduction rate is as follows:

y = (η_oil_ − η_em_)/η_oil_ × 100% (1)

where y is the viscosity reduction rate (%); η_oil_ is the viscosity of crude oil, in mPa·s; η_em_ is the viscosity of the emulsion sample.

(4) Interfacial tension

A TX-500C interfacial tensimeter (TX-500C, USA KINO Industry Co., Boston, MA, USA) was used to measure the interfacial tension. The continuous phase solution was slowly injected into the sample tube until the continuous phase was full of the sample tube, then an oil phase sample of about 1.0 μL was injected into the continuous phase of the sample tube with a microsyringe. The lid of the sample tube was tightly closed and there was no bubble in the sample tube. After preheating, the rotating speed of the test software was adjusted to the set speed of 5000 r/min. The calculation formula of interfacial tension:

σ = ∆p × R_0_^3^×ω^2^/4 (2)

where σ is interfacial tension, mN/m; ∆p is the two phases’ density difference, kg/cm^3^; R_0_ is instrument panel reading speed, m; ω is angular velocity of the sample tube, rad/s.

(5) Wettability Alteration Capacity

An OCA20 contact angle measurement instrument made by Dataphysics (Stuttgart, Germany) was used to perform contact angle measurement and observe the oil film stripping process. The measurements were carried out at ambient conditions according to standard [33].

(6) Oil recovery experiments

Core flooding experiments were conducted to evaluate EOR potential ability. The core was vacuumed, saturated with formation water to determine permeability, and then saturated with crude oil. The specific water permeability, oil permeability, and oil saturation are shown in Table 5. The oil extraction experiment procedure is as follows: the total mineralization of the injected water was 7887 mg/L, with an injection rate of 0.2 mL/min. After water injection was completed, the water content of the produced liquid reached 98%. Then, a 0.5 Pore Volume (PV) viscosity reducer solution was injected to seal the well for three days, and water injection was carried out again until the water content exceeded 98%.

(7) Molecular Simulation

The models of resin and asphaltene, as well as their dimers, were constructed using GhostView v5.0. The structures of resin, asphaltene, and their dimers, along with the interaction configurations and energies between the viscosity reducer and the resin/asphaltene dimers, were calculated using the AM1 method in Gaussian 09. The calculated structures and energies were visualized with Gview.

## 4. Conclusions

In this paper, the performance of microbial-assisted chemical viscosity reducers in improving oil displacement were systematically studied. The results indicated that the optimal combination for microbial-assisted chemical viscosity reduction was OHSB and Microbacterium. The results demonstrated that this new microbial-assisted chemical composite viscosity reducer achieved a viscosity reduction rate exceeding 85% for five different crude oil samples. It effectively altered the wettability of oil-wet surfaces, improved the oil film stripping rate by 50–65% compared to pure chemical flooding agents, and achieved ultra-low oil–water interfacial tension on the order of 10^−3^ mN/m with crude oil, leading to an EOR enhancement of 22–26%. Its oil displacement efficiency was significantly higher than that of a single chemical agent or viscosity-reducing bacterial system.

Microbial-assisted chemical viscosity reduction technology fundamentally represents an upgrade from chemical intervention or biological intervention alone to a novel model of bio-chemical synergistic enhancement. Based on the global energy industry’s pursuit of low cost, high efficiency, and low carbon emissions, this technology should focus on achieving breakthroughs in the following directions. Firstly, it is necessary to promote the refinement and targeted synergistic effect of microorganisms and chemical viscosity reducers, using microbial metabolites to assist chemical viscosity reducers in more effectively penetrating into the interior of asphalt structures, achieving the transition from surface peeling to core disintegration. The second direction is to expand the application potential of this technology in high-temperature, high-salt, deep and ultra-deep heavy oil reservoirs. The third is to enhance functional complementarity by leveraging the in-situ metabolic activity of microorganisms to compensate for the shortcomings of chemical viscosity reducers, such as limited effective distance, high cost, and poor sustainability, while exploiting the high efficiency and immediacy of chemical viscosity reducers to overcome the limitations of slow microbial activation and high environmental susceptibility. In summary, the development of novel microbial-assisted chemical viscosity reduction technology holds broad application prospects and offers a promising new pathway for enhancing the recovery factor of high-viscosity oil reservoirs.

## Figures and Tables

**Table 1:** The viscosity reduction results of different microbial bacteria.

Microbial Bacteria	Viscosity Before Viscosity Reduction/mPa·s	Viscosity After Viscosity Reduction/mPa·s	Viscosity Reduction Rate/%
Acinetobacteria	64.4	17.3	73.1
Dietzia	64.4	25.2	60.9
Microbacterium	64.4	9.5	85.2

**Table 2:** The viscosity reduction results for oils from different oilfields.

Oil Number	Initial Viscosity of Crude Oil/mPa·s	Viscosity After Viscosity Reduction/mPa·s	Viscosity Reduction Rate/%
1#	64.4	9.5	85.2
2#	243	22.8	90.6
3#	1216	36.9	97.0
4#	2720	35.4	98.7
5#	6462	45.2	99.3

**Table 3:** Wettability alteration capacity results of different viscosity reducer systems.

Oil Number	Stripping Oil Film Time/s		
	OHSB System	OHSB-Microbacterium System	Wetting Improvement Ratio/%
1#	23	9	60.87
2#	69	26	62.32
3#	95	41	56.84
4#	103	49	52.43
5#	125	56	55.20

**Table 4:** Interfacial tension results of different viscosity reducer systems.

Oil Number	Interfacial Tension Results/mN·m^−1^	
	OHSB System	OHSB-Microbacterium System
1#	2.3 × 10^−2^	1.9 × 10^−3^
2#	3.1 × 10^−2^	2.3 × 10^−3^
3#	2.9 × 10^−2^	2.5 × 10^−3^
4#	3.7 × 10^−2^	3.2 × 10^−3^
5#	4.1 × 10^−2^	3.6 × 10^−3^

**Table 5:** Oil recovery by viscosity reduction flooding.

Items	1	2	3	4	5
System	Chemical system	Microbial system	Chemical and microbial composite system		
Air permeability (×10^−3^ µm^2^)	243	196	178	189	216
water permeability (×10^−3^ µm^2^)	81	67	61	65	71
Oil Saturation (%, OOIP)	68.72	67.19	66.75	67.02	66.81
Oil recovery by water flooding (%, OOIP)	53.09	51.59	51.36	52.08	51.27
Final oil recovery (%, OOIP)	68.40	63.48	74.03	76.46	76.58
Enhance Oil Recovery (%, OOIP)	15.31	11.89	22.67	24.38	25.31

**Table 6:** Individual and combined energies of resin, asphaltene and OHSB.

Molecular Name	Calculated Energy/Hartree	Combined Energy/kcal/mol
OHSB	−0.40	
resin monomer	−0.35	
resin dimer	−0.71	4.99
resin dimer/OHSB	−1.13	15.88
asphaltene monomer	−0.21	
asphaltene dimer	−0.44	15.60
asphaltene dimer/OHSB	−3.53	33.02

**Table 7:** The characteristics of crude oil samples from various oilfields.

No.	Viscosity of Crude Oil/mPa·s	Oilfield	Reservoir Temperature/°C
1	64.4	Jilin oilfield	30
2	243	Jilin oilfield	32
3	1216	Jilin oilfield	30
4	2720	Xinjiang oilfield	50
5	6462	Xinjiang oilfield	35

## Data Availability

The data presented in this study are available on request from the corresponding author.
